# In Silico Design, Synthesis, and Antibacterial Evaluation of Allyl Esters of Salicylic and Acetylsalicylic Acid and Their Copolymers

**DOI:** 10.3390/molecules30183826

**Published:** 2025-09-21

**Authors:** Eldar Garaev, Namig Rasulov, Shafa Aliyeva, Jamila Yusifova

**Affiliations:** Department of Pharmaceutical Toxicology and Chemistry, Faculty of Pharmacy, Azerbaijan Medical University, Baku AZ1022, Azerbaijan; namiq.resulov.1956@mail.ru (N.R.); alievaashafa@gmail.com (S.A.); camilya@inbox.ru (J.Y.)

**Keywords:** allyl ester of salicylic acid, allyl ester of acetylsalicylic acid, oligoethylene macromonomer, copolymers, copolymerization constants

## Abstract

The main objective of the study was to choose the best salicylic acid-based monomers through in silico research to improve the antibacterial effects of dental prostheses, refine the synthesis process of such monomers, and examine their antibacterial and antifungal properties in vitro, forecast the long-term stability in an oral biological environment using molecular docking software and synthesizing new copolymers. Based on their strong antibacterial activity and low toxicity compared to other derivatives, the allyl ester of salicylic acid (AESA) and the allyl ester of acetylsalicylic acid (AEASA) were chosen as the study objects. Salicylic and acetylsalicylic acids were esterified with allyl alcohol and allyl bromide in a variety of solvents and temperatures to synthesize AESA and AEASA. The optimal conditions were identified with a yield of 78%. IR spectroscopy was used to confirm the chemical structure of synthesized molecules. In the presence of peroxybenzoyl, the regularities of the polymerization process between the obtained monomer and oligoethylene macromonomer (PEMM) were examined. To obtain new antibacterial oligomers containing a salicylic group and to study their physico-chemical properties, a technology for obtaining the copolymers of AESA with PEMM was developed, and their physical, mechanical, and antimicrobial properties were studied.

## 1. Introduction

Antibacterial polymers are an important class of materials for biomedical applications such as dental prostheses, wound dressings, coatings, and implants. Obtaining antibacterial polymer materials by including bactericidal chemicals into their composition is seen as a potential avenue in the development of antibacterial compositions, including dental prostheses. It should be noted that the amount of such additives in polymer mixtures is less than 0.01%. Traditional antibacterial agents often face limitations: risk of bacterial resistance, toxicity, short lifespan of action, and poor stability in biological environments. Therefore, developing new materials that combine antibacterial effectiveness, biocompatibility, low toxicity, and stability is crucial. Antimicrobial additives must meet several conditions, the most important of which are their safety, ease of processing, compatibility with polymers and other additives, lack of a negative impact on polymer physical and mechanical properties, and high efficiency [1]. The goal of incorporating antimicrobial compounds into polymer-based composite materials is to limit the growth of different bacteria on the surface and within polymer products.

Recent research has focused on incorporating salicylic acid derivatives into polymer matrices to make smart antimicrobial materials. For instance, degradable salicylic acid -based polymers have been developed for sustained release of the active moiety, which can provide both high local concentration and prolonged activity [2].

Antimicrobial polymers have been extensively studied for their intrinsic ability to combat pathogenic bacteria, fungi, and viruses. Early synthetic antimicrobial polymers, such as homo- and copolymers of 2-methacryloxytroponones, were inspired by host defense peptides and polymer disinfectants. Subsequent research explored functionalization with salicylic acid derivatives, chitosan, ε-poly-lysine, and cationic polymers such as polyvinyl benzyl ammonium chloride to enhance antimicrobial activity. Mechanistically, cationic polymers interact with negatively charged bacterial envelopes, destabilizing the membrane and increasing permeability, while hydrophobic and cationic interactions are critical for action against *Mycobacterium tuberculosis* and enveloped viruses [3].

In medicine, the copolymers containing salicylates and methyl methacrylate are utilized as part of implants and other applications where adhesion is necessary. Today, numerous antibacterial additives for the copolymers of unsaturated esters of salicylic acid with polyolefins, polystyrene, etc., have been developed and are widely used for this purpose. Incorporating antibacterial additives into base polymers while creating polymer goods or applying an antibacterial layer to a polymer product’s surface are two popular methods for producing antibacterial polymeric materials.

Recent studies in polymer science have emphasized the importance of designing functionalized materials with antibacterial properties to improve their use in biomedicine. Salicylic acid-derived compounds have attracted attention due to their ability to inhibit microbial growth while retaining favorable physicochemical properties. Against this backdrop, the development of copolymers incorporating salicylic acid groups has yielded notable enhancements in structural stability and antimicrobial efficacy [4].

It was found that palmitic acid-salicylate esters are potential active ingredients for antimicrobial drugs and moderate (MIC = 62.5 and 125 mu g/mL) antibacterial and antifungal activity against *Streptococcus pneumoniae*, *Staphylococcus aureus*, *Salomonella typhi*, *Klebsiella pneumoniae*, *Escherichia coli*, *Trichophyton mentagrophytes*, *Microsporum audouinii*, *Epidermophyton floccosum* and *Microsporum gypseum* [5].

The manufacturing of medical devices, such as dental prostheses using the polymers with a biologically active group as an antibacterial additive to prolong the service life is currently of the utmost interest [6].

The purpose of the presented article is an in silico study, optimization of synthesis of antibacterial and toxic properties of the allyl ester of salicylic acid (AESA) and allyl ester of acetylsalicylic acid (AEASA) [7] as the elementary members of the macrochain, obtaining of their new copolymers with oligoethylene macromonomers and studying their antibacterial properties with the aim of making possible the use of these polymers in the production of medical devices, including dental prostheses.

## 2. Results and Discussion

### 2.1. Identification of AESA and AEASA

**Figure 1:**
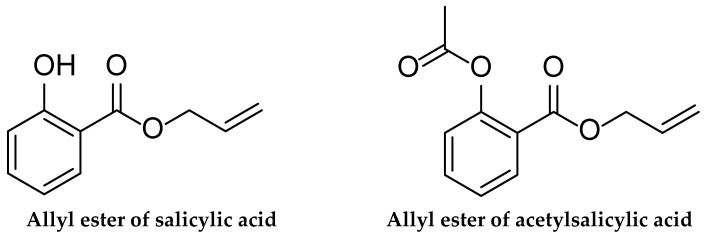
Chemical structure of AESA and AEASA.

The study of the infrared spectra of obtained compounds revealed the following signals: the stretching vibrations of this hydroxyl group cause a broad and strong absorption line in the 3189, 3192, and 3185 cm^−1^ regions of the AESA molecule infrared spectrum. Low-intensity absorption lines that are indicative of the = CH bonds in the aromatic ring of the substance can be seen in the absorption areas of 3064 cm^−1^ and 3030 cm^−1^. The -CH_3_ group exhibits asymmetric stretching vibrations in the absorption region of 2952 cm^−1^, while the -CH_2_ group exhibits symmetric stretching vibrations in the absorption region of 2840 cm^−1^. Strong stretching vibrations associated with the >S = O group are visible in the absorption region at 1672 cm^−1^, while ring vibrations associated with the aromatic ring are visible in absorption regions like 1613, 1586, 1485, and 1384 cm^−1^. In the 1031–1088 cm^−1^ range, symmetrical stretching vibrations associated with the -C(O)-O-CH_2_-(CO-C) bond were detected. The chemical structures of obtained products are presented in Figure 1.

### 2.2. Identification of AESA and PEMM Copolymers

In the IR spectrum of the copolymers, unlike that of oligoethylene spectrum (bands at 749, 1142, 1447, and 2950 cm^−1^ assigned to C–H and CH_2_), 1638 cm^−1^ (C_6_H_4_), characteristic absorption bands are observed at 1142 cm^−1^ (O–CO), 1722 cm^−1^ (C=O), and 3382 cm^−1^ (OH).

**Figure 2:**
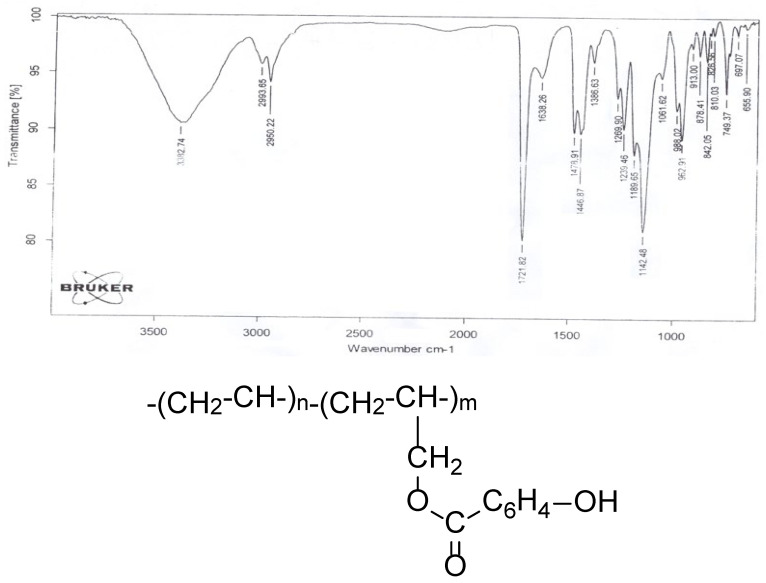
The composition and structure of AESA and PEMM copolymers, according to the results of IR spectra.

According to the results of IR spectra, the composition and structure of the copolymer are as presented in Figure 2.

### 2.3. In Silico Studies

Investigating the biological activity and toxicological characteristics of salicylic acid and its antibacterial derivatives is the primary goal of these studies. Using computer programs like PASS online (https://way2drug.com/PassOnline/, accessed on 15 July 2025), ProTox 3.0, AutoDock vina 4.2, and Swiss Target Prediction (http://www.swisstargetprediction.ch/, accessed on 15 July 2025), it is intended to identify potential antibacterial target proteins of salicylic acid and its derivatives. Additionally, the biological activity and pharmacological properties of these derivatives is assessed, their safety level evaluated by examining the toxicity profiles of ProTox 3.0, and receptor-ligand interactions are studied using the molecular docking method [8,9]. These studies are crucial for establishing a solid scientific foundation for the development of novel antibacterial products and for conducting a thorough evaluation of the therapeutic potential of derivatives of salicylic acid.

The PASS Online program was used to ascertain the antibacterial properties of salicylic acid and its derivatives, as well as the differences in the biological activity of AESA and AEASA (Table 1).

Comparing the antibacterial properties of salicylic acid and its derivatives, it was determined by the PASS Online program that, although salicylic acid has an antibacterial property of 40%, AESA has 23%, AEASA has 37%, methacrylic ester of salicylic acid has 33%, and the value of this parameter of acryl ester of salicylic acid is 35%. Consequently, the allyl esters of salicylic acid and acetylsalicylic acid were selected as the subject of study based on these and additional indicators.

The study’s next phase involved conducting in silico assessments of two salicylic acid derivatives (AESA and AEASA) using the SwissTargetPrediction tool. In terms of clarifying the derivatives’ antibacterial processes and locating possible target biomolecules, the data obtained offer crucial information.

A sophisticated in silico analysis tool for predicting the toxicological profile of bioactive substances is the ProTox 3.0 platform. This tool evaluates the molecule’s LD50 (lethal dose), organ-specific toxicity, mutagenesis potential, carcinogenic effect, and immunotoxicity to provide a high-precision assessment of its likely harmful consequences.

AESA has low toxicity, according to the analysis carried out with the ProTox 3.0 program:

- The substance’s LD50 of 2.3 g/kg suggests that it presents a modest danger risk.
- Toxicity Class: Class 5—Substances in this class are not very dangerous and have a modest potential for toxicity.
- Average Similarity: 59.74%—The average similarity number indicates how examined compound structurally and physically are close to known hazardous compounds.
- Prediction Accuracy: 67.38%—The program’s reliability for this test is of medium level.

The following findings can be drawn from the analysis done using the ProTox 3.0 program:

- Because of its low toxicity, the chemical may be a viable medication candidate.
- Its safe dosage can be ascertained using the LD50 value.
- The substance’s lipophilicity, indicated by its LogP value of 2.57, implies that it has a good chance of crossing biomembranes.

According to AEASA’s analysis using ProTox 3.0, moderate toxicity is predicted:

- The substance’s LD50 of 1.2 g/kg suggests that it presents a medium danger risk.
- Toxicity Class: Class 3—Substances in this class are not very dangerous and have a modest potential for toxicity.
- Average similarity: 81.07%—this indicates that the substance shows similarity to some known toxic substances, but is not considered to be completely toxic.
- Prediction Accuracy: 70.97%—The reliability of the analysis results is of medium level.

The following findings were indicated by ProTox 3.0 program:

- Because of its low toxicity, it may be a viable medication candidate.
- logP (1.95): Enhances the material’s lipophilicity, which may have a favorable impact on its biological activity by enabling it to flow through cell membranes.

### 2.4. Molecular Docking

The primary objective of this study is to investigate the interactions between selected compounds (ligands) and the oral cavity enzyme amylase. For this purpose, the “.pdb” format of the amylase enzyme was retrieved from the Protein Data Bank (https://www.rcsb.org/, accessed on 15 July 2025) (PDB ID: 1UD2). In this research, the docking interactions of amylase protein with the selected ligands were examined, and their binding characteristics were evaluated. Docking analyses were carried out to identify the potential binding sites of the amylase protein and to elucidate the interaction patterns of ligands within these sites.

Protein Preparation Stage:

The crystal structure of the amylase enzyme was obtained and refined using Discovery Studio Visualizer 21.1.0.20298 (https://discover.3ds.com/discovery-studio-visualizer-download, accessed on 15 July 2025) by removing non-essential molecules, such as ligands and water. The protein was then prepared for subsequent processes with AutoDock Tools Version 1.4.5 (https://autodock.scripps.edu/, accessed on 15 July 2025), where polar hydrogen atoms and Kollman charges were added. Finally, the three-dimensional structure of the protein was saved in the “pdbqt” format.

Ligand Preparation Stage:

The preparation of ligands is a crucial step for the successful execution of docking analyses in AutoDock Vina. Initially, the three-dimensional (3D) structural models of the ligands were obtained using Chemaxon Marvin 24.3.0—Chemical Drawing Software (https://chemaxon.com/marvin, accessed on 15 July 2025). Subsequently, the structures were converted into the “.pdbqt” format using AutoDock Tools Version 1.4.5. As a result, the optimal conformations of the ligands were obtained for simulation purposes.

Using AutoDock Tools Version 1.4.5, the grid box coordinates were selected in the following format: dimensions of 40 × 40 × 40 with a spacing of 0.375 Å, centered at X: 9.479, Y: 83.805 and Z: 99.866.

Using AutoDock Vina 4.2 software, a molecular docking analysis of two distinct ligands with the amylase enzyme was carried out in this study. The findings indicate that ligands have a weak propensity to attach to the amylase enzyme, with free energy values of −5.6 and −5.4 kcal/mol, respectively. It should be mentioned that it is better if the ligands have little interaction with oral enzymes because the primary objective of the study was to choose appropriate ligands to improve the antibacterial qualities of oral prostheses. Strong binding to enzymes hence enhances the ligand’s susceptibility to biotransformation and degradation, which may shorten the antibacterial action’s duration and decrease its stability on the prosthesis surface.

The modest contact of the two chosen ligands with the amylase enzyme is indicated by the binding energy values, which is thought to be a significant factor in their long-term activity in the oral cavity. They can specifically catalyze the breakdown of certain hydrolytic enzymes in the oral environment, such as proteins, amylase, and low-molecular substances. Nonetheless, the ligands’ comparatively low binding indices suggest that they are less likely to be broken down by enzymes, which would help maintain their potent antibacterial properties on prosthesis surfaces. Furthermore, additional in vitro and in vivo studies are required to ascertain the impact of ligands on oral microbiota and their long-term antibacterial effectiveness.

The antibacterial qualities of the ligands to be utilized here are crucial, but so are their biocompatibility and toxicological profile, taking into account the particular oral environment, pH variations, enzyme activity, and saliva composition. The long-term stability of chosen chemicals and their optimal bond strength with the dental prosthesis material can be guaranteed by minimal contact with oral enzymes. In this sense, it is critical to assess the docking results not just in terms of the bond energy but also the ligand’s potential behavior in the oral bioenvironment, as well as its diffusion capacity and biostability. Overall, the results of the analyses indicate that these ligands can be considered as viable options for usage in the oral cavity; however, additional studies are required to verify their stability and efficacy in real settings.

#### 2.4.1. Interaction Between the Enzyme Amylase and AESA

**Figure 3:**
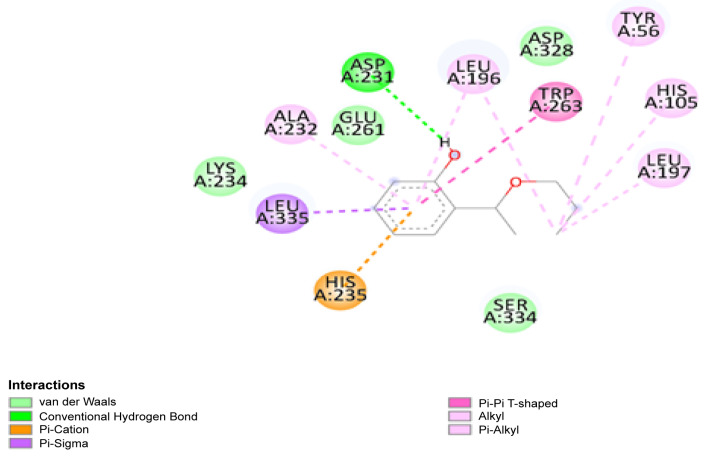
Two-dimensional structure of the docking results of AESA with amylase enzyme.

Due to the results of molecular docking analysis, it was found that the highest binding energy of AESA with the enzyme amylase was −5.6 kcal/mol (Figure 3). This indicates that the ligand has a moderately weak interaction with the enzyme. If this ligand is intended to be used as an antibacterial component in oral prostheses, its low binding energy with the enzyme can be considered a positive factor.

**Figure 4:**
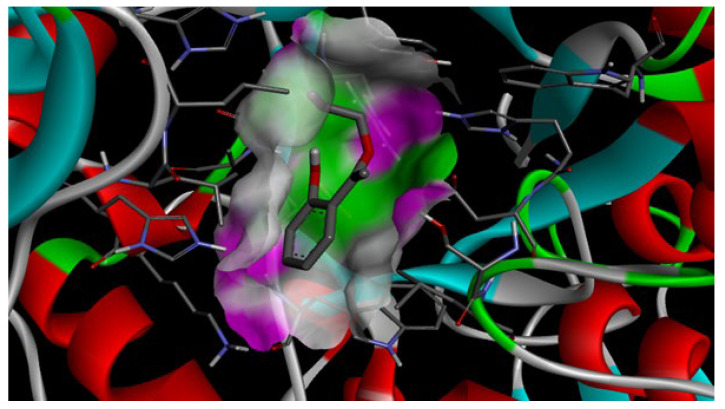
Three-dimensional structure of the docking results of AESA with amylase enzyme.

The interaction of AESA with amylase is important to maintain its stability on the prostheses surface. If the binding energy was higher (e.g., around −7 or −8 kcal/mol), it would indicate a stronger binding of the ligand to the active site of the enzyme, which would affect the enzyme’s activity (Figure 4). The value of −5.6 kcal/mol, however, suggests that AESA has a poor capacity to bind precisely to the enzyme’s active site and can withstand longer stability in the oral bioenvironment.

#### 2.4.2. Interaction Between AEASA and the Amylase Enzyme

**Figure 5:**
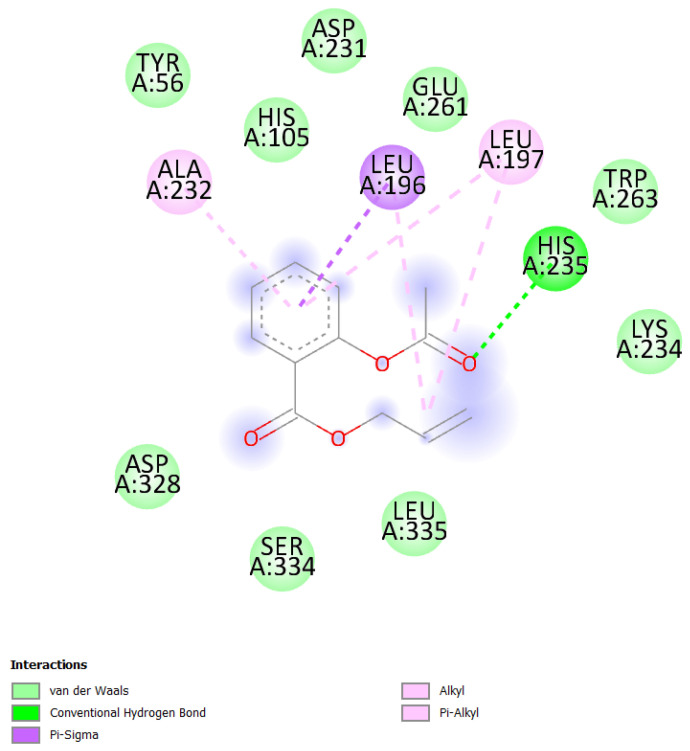
Two-dimensional structure of the docking results of AESA with amylase enzyme.

In this context, AutoDock Vina 4.2 software was used to evaluate the binding tendency of AEASA with the amylase enzyme. and −5.4 kcal/mol was found to be the obtained binding free energy value (Figure 5).

**Figure 6:**
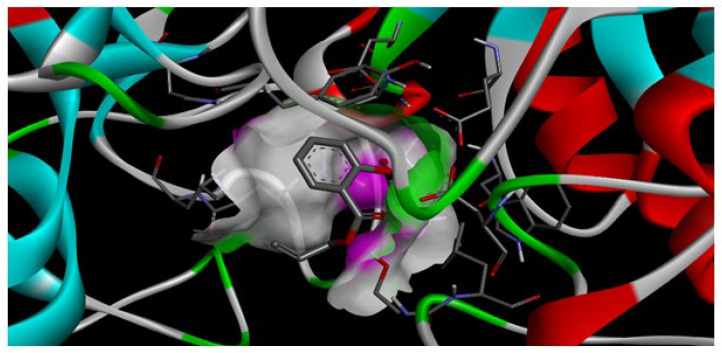
Three-dimensional structure of the docking results of AEASA with amylase enzyme.

The low binding energies of AEASA with the amylase enzyme suggest that they do not establish a significant connection with the enzyme’s active core, based on the results of the molecular docking (Figure 6). This can guarantee that the enzyme’s antimicrobial activity will last for a long time on the surface of prostheses without compromising the normal function of the enzyme.

As a result, it should be mentioned that the interaction of AEASA with the enzyme amylase suggests that it could be a good option for oral cavity prosthetic materials that have antibacterial properties. To learn more about this compound’s interactions with other oral enzymes (lysozyme, proteases), adhesive stability on the prosthesis surface, and biocompatibility under real settings, more in vitro and in vivo studies are necessary.

### 2.5. Synthesis of AESA and PEMM

Studies have shown that the AESA monomer possesses stronger antibacterial properties compared to AEASA. However, in dental prostheses, polymers are required to exhibit lower antibacterial activity. Therefore, we decided to obtain the copolymer of AESA with PEMM. One of the most often used and produced polymers is polyethylene (PE). It can be applied in a variety of fields due to its mechanical and physicochemical characteristics. Nevertheless, PE’s poor miscibility with other polymers prevents the creation of homogenous composite materials and composites. It is crucial from a scientific and practical standpoint to eliminate this drawback of PE by its functionalization. This way, PE may be used to produce more expensive goods and broaden its range of applications. PE’s weak resistance to microbial action is one of its drawbacks; items manufactured of it, particularly tools, equipment, and packaging materials that are frequently used in the food and medical industries, soon lose their usefulness or even start to harbor microorganisms.

PE can be functionalized in two ways: either by adding additional active functional groups to oligoethylenes that include unsaturated bonds at the end group, or by copolymerizing with other physiologically active vinyl monomers because of the vinyl group at the end of the macromolecule. In the latter approach, the reaction mostly produces block copolymers. This type of functionalization is a novel kind of modification technique that is distinct from other techniques.

To obtain new antibacterial oligomers containing a salicylic group and to study their physicochemical properties, a technology for obtaining the copolymers of allyl ester of salicylic acid with polyethylene macromers was developed, and their physical, mechanical and antimicrobial properties were studied.

**Figure 7:**
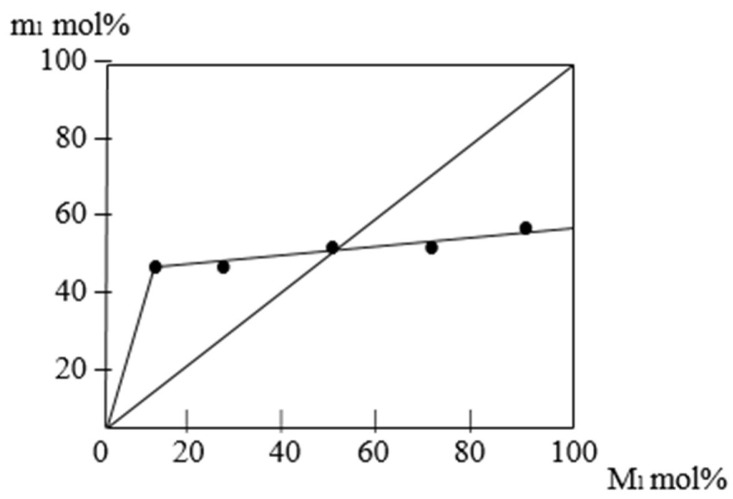
Graph of the dependence of the composition of copolymers in the copolymerization reactions of AESA with PEMM on the composition of the monomers taken for the reaction as presented in Table 2.

The low prevalence of allyl monomers as raw materials for the synthesis of synthetic polymeric materials can be attributed to their poor rate of polymerization by the radical process and the low molecular weight of the resulting polymer products. Since allyl monomer units can be incorporated into the macromolecules of well-known polymers to create modified polymeric materials with specific mechanical and physical properties, particular attention is given to the copolymerization of allyl compounds with other monomers in this context. However, because allyl compounds undergo a chain transfer reaction, monomers containing an allyl group also have a weak propensity to copolymerize due to their structure. A new radical is created when the hydrogen in the allyl group splits off during the growth of a polymer chain. This radical is stabilized by resonance with the nearby double bond in the monomer. As a result, the freshly generated radical creates a less stable radical by binding to the subsequent monomer molecule. Using the donor-acceptor mechanism in the presence of peroxide initiators is one of the efficient ways to include monomers with an allyl group in the copolymerization reaction. The following outcomes were achieved from the copolymerization reaction of oligoethylene macromonomers with AESA for this purpose:

Table 2 and Figure 7 demonstrate that the AESA monomer (M1) has a somewhat higher relative activity than PEMM in the copolymerization of the specified monomers. This is because high molar mass alpha-olefins do not participate in radical polymerization. The PEMM monomer’s Alfrey–Price parameters (Q = 3.2 and e = 0.2) were determined. PEMM is a passive monomer, according to the particular activity value. On the other hand, PEMM’s polarity factor (e) value is likewise near zero. In this case, because the comonomer and the allyl ester have similar polarity, the comonomers are less likely to undergo copolymerization processes. Consequently, a change in the reaction mixture’s comonomer ratio (an increase in PEMM quantity) alters the reaction rate as well as the molecular weight of the copolymers that are produced.

**Figure 8:**
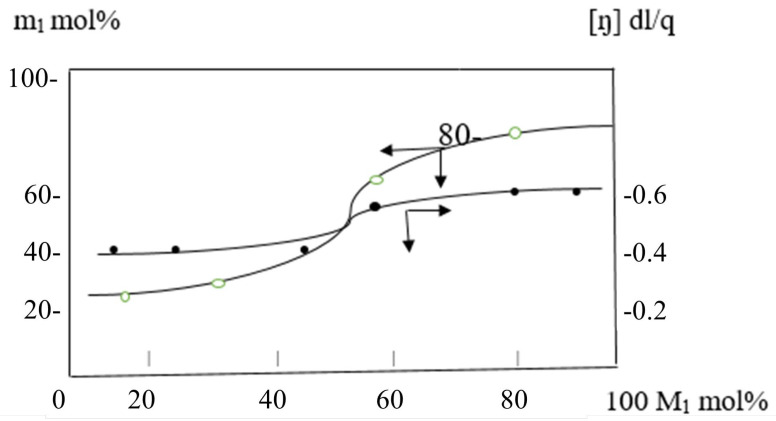
The increase in molar ratio of PEMM in the monomer mixture increases the rate of the copolymerization reaction.

Figure 7 shows once more that the copolymers do not undergo a radical homopolymerization reaction since the quantity of PEMM in them is less than 50 mol% and the amount of AESA is nearly 50%. Both PEMM and AESA enter only copolymerization reactions. This indicates that an increase in the amount of PEMM and AESA in the monomer mixture results in a decrease in the molecular weight of the copolymer because both of them actively participate in the chain transfer reaction through the monomer (Figure 8).

Figure 8 inherent viscosity of the copolymers produced in the copolymerization reactions of AESA with PEMM, as well as how the reaction yield varies depending on the original monomer mixture’s composition.

### 2.6. The Study of Antibacterial Properties of AESA and AEASA

The serial dilution approach was used to investigate the antibacterial qualities of AESA and AEASA, which are the monomers of synthesized copolymers.

The following table lists the antibacterial activity of synthesized compounds and controls:

According to the results presented in Table 3, the synthesized substances had various effects on different microorganisms. In particular, when diluted in a ratio of 1:100, effective antimicrobial activity was observed against all tested microorganisms.

These compounds’ antibacterial activity was examined in comparison to phenol and nitrofungin, two common antiseptics used in medicine (Table 4).

While other compounds exhibited antibacterial action against the identical test cultures, only phenol outperformed the other control chemicals in terms of its effect on blue-green pus forming bacteria.

AEASA exhibits the strongest antibacterial activity among these findings. As a result, AESA (I) was more successful against blue-green pus forming bacteria and Candida fungi. Candida was destroyed in only 20 min with a 1:400 dilution, whereas *E. coli* and blue-green pus forming bacteria were killed in 10 min. At a 1:700 dilution, however, *S. aureus* was only killed in 20 min, and at other dilutions, no effect was seen for an hour. It is interesting to see that *C. fungus* was destroyed in 40 min by a 1:800 dilution.

At a 1:100 dilution, AEASA (II) destroyed *C. fungus* in 10 min and *S. aureus* in 40 min.

Cliform bacteria (*E. coli*) and blue-green pus forming bacteria (*P. aeruginosa*) were totally killed in 20 min.

It was discovered that, based on their antibacterial action, monomers with a salicylic group can be prioritized as follows: Acid salicylic > AEASA > AESA.

The bactericidal qualities of copolymers of oligoethylene macromonomer with AEASA in different ratios were studied. The copolymers and their sodium salts were tested in physiological solutions for their Minimal Inhibitory Activity (MIA) against *S. aureus*, *P. aeruginoza*, *C. albicans*, and *E. coli* (Table 5).

Copolymers with 5–10 weight percent of the elementary component AEASA were found to have antibacterial qualities that are two to three times greater than those of the oligoethylene macromonomer.

## 3. Materials and Methods

### 3.1. Synthesis of AESA

The esterification reaction with alcohols and unsaturated halogenated chemicals is the primary process for producing salicylic acid compound esters [10].

The starting reagents were introduced into a 100 mL three-neck flask with 6.9 g (0.05 mol) of salicylic acid, 7.4 g (13 mL 0.1 mol) of allyl bromide, and 8.125 × 10^−4^ g (5 × 10^−6^ mol) of FeCl_3_ as a catalyst and 20 mL of dried benzene. Water was then distilled out by attaching a reflux condenser to the flask. There were no adverse effects from the reaction, and the catalyst activity did not drop. The highest yield of the product (78%) was obtained when the reaction was conducted for 6 h at the boiling temperature. The reaction mixture was first cleaned with water to get rid of extra alcohol and solvent after cooling. It was further refined by straightforward distillation, which involved removing the ester entirely from the mixture using a water pump. After the solvent had evaporated, the reaction product was concentrated in a water vacuum, and the 125–127 °C fraction was isolated.

### 3.2. Synthesis of AEASA

A three-necked flask with a ball condenser, separatory funnel, and thermometer was filled with 18.3 g of salicylic acid, K_2_CO_3_, and 100 mL of benzene. After stirring for 30 min, 2 mL of pure sulfuric acid was added. Over the course of 20 min, 32 mL of allyl alcohol was added to the mixture via a separatory funnel. The resultant solution was heated to 60–70 °C with stirring for 3 h. The reaction was then stopped at the next stage, and the product was transferred to a Claisen flask. The unreacted part of the allyl alcohol was evaporated in the Claisen flask, which was furnished with a receiver, an allonge, and a Liebig condenser. The unreacted part of the salicylic acid and ethyl alcohol dissolved by placing the residue in a chemistry glass of cold water. Na_2_CO_3_ was added to the mixture, which creates a slightly alkaline medium, and allyl ester of acetylsalicylic acid was separated. Diethyl ether is used in a separatory funnel to extract the final product [11].

### 3.3. Synthesis of AESA and PEMM Copolymers

The radical polymerization approach was used to perform copolymerization reactions between oligoethylene macromonomers and allyl esters of salicylic and acetylsalicylic acids [12].

PEMM, used for copolymerization, was obtained by thermal destruction of low-density polyethylene (LDPE) under special conditions. The average molecular weight is approximately 400 and the degree of polydispersity is 1.08. This copolymer is made by adding a reaction mixture to a 100 mL glass ampoule that contains 1.5 g AESA, 13.5 g PEMM, 0.035 g benzoyl peroxide (0.3%), and 30 mL methyl ethyl ketone. After cooling the ampoule and removing any remaining air with a stream of nitrogen gas, the neck is welded shut and heated to 70 °C for 8 h in a thermostat. After precipitating the solid polymer solution from the ethanol, it is cleaned with ester and dried at 40 °C in a vacuum drier until reaching the required mass. The yield is 72%, or 10.8 g. The resulting copolymer is a white powder that dissolves easily in acetone, chloroform and dioxane. When heated, it becomes partially soluble in Na_2_CO_3_ solution [13].

### 3.4. Spectroscopic Analysis

Using an Alfa Bruker spectrometer (Billerica, MA, USA), the produced copolymers’ infrared spectra were captured in the 600–4000 cm^−1^ range (KBr sheet, ATR). Used background absorption. Number of scan was selected 6. The calibration method was using a polystyrene film standard.

### 3.5. In Silico Studies

In silico studies were conducted using such software as PASS online (https://www.way2drug.com, accessed on 15 July 2025], ProTox 3.0 (https://tox.charite.de/protox3, accessed on 15 July 2025) and Auto Dock Vina 4.2. [https://autodock.scripps.edu, accessed on 15 July 2025].

### 3.6. Antibacterial Evaluation

The serial dilution approach was used to examine the antibacterial activity of synthetic compounds [13].

To do this, sterile distilled water was used to dilute 1% solutions of produced compounds in ethyl alcohol (1:100, 1:200, 1:400, and 1:800). The number of repetitions was two for each dilution. The antimicrobial properties of the substances were examined in comparison to ethanol, phenol, and nitrofungin. Test cultures of Gram-positive bacteria (*Staphylococcus aureus,* ATCC 28213), Gram-negative coliform bacteria (*Escherichia coli,* ATCC 25922), blue -green pus forming clinical strain pigment bacteria (*Pseudomonas aeruginosa*), and clinical strain Candida fungi (*Candida albicans*), were used. Bacteria were cultivated on MPA (meat-peptone agar) media, whereas fungi were cultivated on Sabouraud Agar. The pH is adjusted to around 5.6. As control were used dilutent [14].

The cultures were inoculated every 10, 20, 40, and 60 min. The cultures were maintained in a thermostat at 37 °C for 24 h for bacteria and 48 h at 28 °C for fungi. Each test tube received one or two drops of the emulsion containing 500 million microorganisms in 1 milliliter throughout the experiment. After 10, 20, 40, and 1 h, the cultures were taken from each test tube inoculated.

## 4. Conclusions

In this study, the allyl esters of salicylic acid (AESA) and acetylsalicylic acid (AEASA) were successfully synthesized, and AESA was copolymerized with oligoethylene macromonomers (PEMM). They were then evaluated for their antibacterial properties through in silico and in vitro analyses. PASS online, ProTox 3.0, and AutoDock Vina tools revealed that both AESA and AEASA possess moderate to strong antibacterial potential and acceptable toxicity profiles, indicating their suitability for biomedical applications, particularly in dental prosthetics.

The main challenge in this research study was to find polymers that will not react with salivary amylase and will maintain antibacterial properties and appropriate solidity for medical use.

The copolymerization of AESA with PEMM demonstrated that the inclusion of salicylic acid derivatives significantly enhanced the antibacterial activity of the resulting polymers, without compromising their physical and mechanical properties. Molecular docking results showed weak binding energies with amylase enzyme, suggesting prolonged stability and sustained antimicrobial effects in the oral cavity.

Experimental data confirmed the synthesized compounds’ bactericidal and fungicidal activity against *S. aureus*, *E. coli*, *Pseudomonas aeruginosa*, and *Candida albicans*, showing comparable or superior performance to some conventional antiseptics.

The next step of our findings is to check ADMET properties of AESA and AEASA-based copolymers in vivo.

These findings highlight the potential of AESA and AEASA-based copolymers as promising candidates for the development of long-lasting, biocompatible antibacterial polymeric materials for medical use.

The copolymer of AESA with PEMM exhibits antibacterial properties. In addition, in silico analysis indicated its low toxicity (Toxicity Class 5). The binding energy value of –5.6 kcal/mol suggests that AESA has a limited capacity to bind specifically to the active site of amylase yet demonstrates prolonged stability in the oral bio-environment. These properties open up new possibilities for the use of the copolymer in the fabrication of dental prostheses.

## Figures and Tables

**Table 1:** The differences in antibacterial properties of salicylic acid and its derivatives.

	Salicylic Acid		Allyl Ester of Salicylic Acid		Allyl Ester of Acetylsalicylic Acid	
	Pa	Pi	Pa	Pi	Pa	Pi
Antibacterial	0.404	0.029	0.236	0.091	0.378	0.036

**Table 2:** Indicators for calculating the relative activities of monomers in the copolymerization reactions of AESA (M1) and PEMM (M2).

Composition of the Initial Monomer Mixture. mol% M1:M2	Output %	Copolymer Composition. mol%		r1	r2	r1r2
		m1	m2			
10:90	5.8	50.23	49.77			
25:75	6.3	50.63	49.37			
50:50	8.0	51.85	49.15	1.2	0.01	0.012
75:25	5.0	53.47	47.53			
90:10	4.8	54.71	45.29			

(Solvent—dioxane, initiator—benzoyl peroxide (0.3%), reaction time—4 h, T = 70 °C).

**Table 3:** Antimicrobial activity of AESA and AEASA.

Test Cultures	Exposure Time (min)	Synthesized Substances							
		Allyl Ester of Salicylic Acid				Allyl Ester of Acetylsalicylic Acid			
		1	2	3	4	1	2	3	4
*S. aureus*	10	-	-	+	+	-	-	+	+
	20	-	-	+	+	-	-	+	+
	40	-	-	+	+	-	-	-	+
	60	-	-	+	+	-	-	-	+
*P. aeruginoza*	10	-	+	+	+	-	-	-	+
	20	-	-	+	+	-	-	-	+
	40	-	-	+	+	-	-	-	+
	60	-	-	+	+	-	-	-	+
*E. coli*	10	-	+	+	+	-	-	-	+
	20	-	-	+	+	-	-	-	+
	40	-	-	+	+	-	-	-	-
	60	-	-	+	+	-	-	-	-
*C. albicans*	10	-	-	+	+	-	-	+	+
	20	-	-	+	+	-	-	-	+
	40	-	-	+	+	-	-	-	+
	60	-	-	+	+	-	-	-	+

Legends: “+” indicates complete inhibition, “-” indicates no inhibition. The dilution factors are denoted by 1 (1:100), 2 (1:200), 3 (1:400), and 4 (1:800).

**Table 4:** Antimicrobial activity of control agents (nitrofungin and phenol).

Test Cultures	Exposure Time (min)	Control Substances							
		Phenol				Nitrofungin			
		1	2	3	4	1	2	3	4
*S. aureus*	10	-	+	+	+	NT	NT	NT	NT
	20	-	+	+	+	NT	NT	NT	NT
	40	-	+	+	+	NT	NT	NT	NT
	60	-	+	+	+	NT	NT	NT	NT
*P. aeruginoza*	10	+	+	+	+	NT	NT	NT	NT
	20	-	+	+	+	NT	NT	NT	NT
	40	-	+	+	+	NT	NT	NT	NT
	60	-	+	+	+	NT	NT	NT	NT
*E. coli*	10	-	+	+	+	NT	NT	NT	NT
	20	-	+	+	+	NT	NT	NT	NT
	40	-	+	+	+	NT	NT	NT	NT
	60	-	+	+	+	NT	NT	NT	NT
*C. albicans*	10	+	+	+	+	-	-	+	+
	20	+	+	+	+	-	-	-	+
	40	-	+	+	+	-	-	-	+
	60	-	+	+	+	-	-	-	+

Legends: The dilution factors are denoted by 1 (1:100), 2 (1:200), 3 (1:400), and 4 (1:800). A “+” denotes total inhibition, or the total eradication of germs; a “-” denotes no inhibition; a “NT” denotes not tested.

**Table 5:** Antimicrobial activity of AEASA and PEMM copolymers.

Test Cultures	Exposure Time (min)	Synthesized Copolymer							
		PEMM				Sopolimer ASTAE-PEMM			
		1	2	3	4	1	2	3	4
*S. aureus*	10	-	-	-	+	-	-	+	+
	20	-	-	-	+	-	-	+	+
	40	-	-	-	-	-	-	-	+
	60	-	-	-	-	-	-	-	-
*P. aeruginoza*	10	-	-	-	+	-	-	-	+
	20	-	-	-	+	-	-	-	+
	40	-	-	-	+	-	-	-	+
	60	-	-	-	+	-	-	-	-
*E. coli*	10	-	-	-	+	-	-	+	+
	20	-	-	-	+	-	-	-	+
	40	-	-	-	+	-	-	-	-
	60	-	-	-	-	-	-	-	-
*C. albicans*	10	-	-	-	+	-	-	-	+
	20	-	-	-	-	-	-	-	+
	40	-	-	-	-	-	-	-	+
	60	-	-	-	-	-	-	-	-

The dilution factors are denoted by 1 (1:100), 2 (1:200), 3 (1:400), and 4 (1:800).

## Data Availability

The original contributions presented in this study are included in the article. Further inquiries can be directed to the corresponding author(s).

## Abbreviations

The following abbreviations are used in this manuscript:

Abbreviation	Full Term
AESA	Allyl Ester of Salicylic Acid
AEASA	Allyl Ester of Acetylsalicylic Acid
PEMM	Polyethylene Macromonomer
IR	Infrared Spectroscopy
LogP	Logarithm of Partition Coefficient
MIA	Minimal Inhibitory Activity
*S. aureus*	*Staphylococcus aureus*
*P. aeruginosa*	*Pseudomonas aeruginosa*
*E. coli*	*Escherichia coli*
*C. albicans*	*Candida albicans*
